# Temporal Dynamics of Glyoxalase 1 in Secondary Neuronal Injury

**DOI:** 10.1371/journal.pone.0087364

**Published:** 2014-02-03

**Authors:** Philipp Pieroh, Marco Koch, Daniel-Christoph Wagner, Johannes Boltze, Angela Ehrlich, Chalid Ghadban, Constance Hobusch, Gerd Birkenmeier, Faramarz Dehghani

**Affiliations:** 1 Department of Anatomy and Cell Biology, Martin Luther University Halle-Wittenberg, Halle (Saale), Germany; 2 Institute of Anatomy, University of Leipzig, Leipzig, Germany; 3 Fraunhofer Institute for Cell Therapy and Immunology, Leipzig, Germany; 4 Translational Centre for Regenerative Medicine, Leipzig, Germany; 5 Massachusetts General Hospital and Harvard Medical School, Boston, Massachusetts, United States of America; 6 Institute of Biochemistry, University of Leipzig, Leipzig, Germany; University of Iowa, United States of America

## Abstract

**Background:**

Enhanced glycolysis leads to elevated levels of the toxic metabolite methylglyoxal which contributes to loss of protein-function, metabolic imbalance and cell death. Neurons were shown being highly susceptible to methylglyoxal toxicity. Glyoxalase 1 as an ubiquitous enzyme reflects the main detoxifying enzyme of methylglyoxal and underlies changes during aging and neurodegeneration. However, little is known about dynamics of Glyoxalase 1 following neuronal lesions so far.

**Methods:**

To determine a possible involvement of Glyoxalase 1 in acute brain injury, we analysed the temporal dynamics of Glyoxalase 1 distribution and expression by immunohistochemistry and Western Blot analysis. Organotypic hippocampal slice cultures were excitotoxically (N-methyl-D-aspartate, 50 µM for 4 hours) lesioned in vitro (5 minutes to 72 hours). Additionally, permanent middle cerebral artery occlusion was performed (75 minutes to 60 days).

**Results:**

We found (i) a predominant localisation of Glyoxalase 1 in endothelial cells in non-lesioned brains (ii) a time-dependent up-regulation and re-distribution of Glyoxalase 1 in neurons and astrocytes and (iii) a strong increase in Glyoxalase 1 dimers after neuronal injury (24 hours to 72 hours) when compared to monomers of the protein.

**Conclusions:**

The high dynamics of Glyoxalase 1 expression and distribution following neuronal injury may indicate a novel role of Glyoxalase 1.

## Introduction

Methylglyoxal (MG) is a metabolite derived from ketone and threonine metabolisms, but is mainly formed non-enzymatically from triose phosphates degradation along the glycolytic pathway [1]–[3]. It was reported that MG exerts cytotoxic effects through induction of reactive oxygen species (ROS), DNA damage, apoptosis, protein modifications and depletion of adenosine triphosphate (ATP) and glutathione (GSH) [4]–[6].

Approximately 0.1–0.4% of the glucotriose flux leads to the formation of MG. Under anaerobic conditions enhanced glycolysis occurs and an increased amount of MG is formed reaching cytotoxic concentrations [7].

In addition, increased MG levels were associated with elevated release of interleukin 1β (IL-1β) and tumour necrosis factor α (TNF-α) in primary neuronal cultures [8], [9]. Neurons show a reduced capacity to adopt their glycolytic rate to anaerobic conditions and are therefore highly susceptible to MG [2], [10].

The detoxification of this harmful metabolite is assured by the ubiquitous glyoxalase system. The system consists of glyoxalase 1 (Glo1) and glyoxalase 2 (Glo2), which convert MG to the non-toxic D-lactate in the presence of GSH [7], [11]. Glo1, a dimeric metalloenzyme with a molecular mass of 46 kDa, was detected in human brain and bovine endothelial cell cultures [2], [12], [13]. However, little is known about the significance of Glo1 following neuronal lesions. Hence, in the present study we analysed the temporal dynamics of Glo1 expression and distribution in secondary neuronal injury by using the *in vitro* model of excitotoxically-lesioned rat organotypic hippocampal slice cultures (OHSC) and the *in vivo* model of permanent middle cerebral artery occlusion (pMCAO) in rats.

## Materials and Methods

### Ethics statement

All animal experiments were performed in accordance with the Policy on Ethics and the Policy on the Use of Animals in Neuroscience Research as indicated in the directive 2010/63/EU of the European Parliament and of the Council of the European Union on the protection of animals used for scientific purposes and were approved by the local authorities for care and use of laboratory animals (State of Saxony: internal reference number TVV 18/07). All surgery was performed under deep anaesthesia (see below), and all efforts were made to minimize suffering.

### Materials and chemicals

For the described experiments the following materials and chemicals were used: Minimal essential medium (MEM), glutamine, Hanks' Balanced Salt Solution (HBSS), normal horse serum (NHS; Gibco BRL Life Technologies, Eggenstein, Germany), glucose (Braun, Melsungen, Germany), cell-culture inserts with a pore size of 0.4 µm (Millipore, Schwalbach/Ts., Germany), vibratome (Vibratom VT 1200 S; Leica Microsystems AG, Wetzlar, Germany), six-well culture dishes (Falcon, BD Biosciences Discovery Labware, Bedford, MA, USA), streptomycin, penicillin, ascorbic acid, N-methyl-D-aspartate (NMDA; Sigma-Aldrich, Deisenhofen, Germany), insulin (Boehringer, Mannheim, Germany), ketamine hydrochloride (Merial, Hallbergmoos, Germany), xylacin (Bayer, Leverkusen, Germany, atropine (Ratiopharm, Ulm, Germany), bicinchoninic acid (BCA) test, Tris (Roth, Karlsruhe, Germany), Glycerol (Sigma-Aldrich), β-mercaptoethanol (Sigma-Aldrich, Steinheim, Germany), bromophenol blue (AppliChem, Darmstadt, Germany), chemiluminescence, superfrost microscope slides from (Thermo Fisher Scientific, Rockford, IL, USA), cryostat 3050 S (Leica), coated slides (Menzel, Braunschweig, Germany), X-ray films (Kodak, Stuttgart, Germany), Dako fluorescent mounting medium (Dako, Hamburg, Germany), Entellan (Merck, Darmstadt, Germany)

#### Antibodies

The following antibodies were used: mouse monoclonal antibody against mouse Glo1 (cat.-No #02-15, clone 2F7, BioMac GmbH, Leipzig, Germany), beta-actin (cat.-No A1978), biotinylated goat anti-mouse IgG (cat.-No B-7264) and ExtrAvidin-horseradish peroxidase (cat.-No E2886) from Sigma, St. Louis, MO, USA), horseradish peroxidase anti-mouse IgG (cat.-No PI-2000, Vector Laboratories, Burlingame, CA, USA), rabbit polyclonal antibody against mouse neuronal specific nuclear protein (NeuN; cat.-No abn78, Millipore, Billerica, USA), rabbit polyclonal antibody against cow glial fibrillary acidic protein (GFAP; cat.-No Z0034) and rabbit polyclonal antibody against rat laminin (cat.-No Z0097; Dako), rabbit anti-human N-Cadherin (cat.-No ab18203, abcam, Cambridge, UK), rabbit anti-mouse ionized calcium binding adaptor molecule 1(Iba1; cat.-No 019-19741, Wako, Osaka, Japan), Alexa fluor dye 488 (goat anti-mouse IgG, cat.-No A-11029) and Alexa fluor dye 568 (goat anti-rabbit IgG, 1∶500, cat.-No A-11036) from Invitrogen, Karlsruhe, Germany.

### Tissue handling and experimental design

#### Organotypic hippocampal slice cultures (OHSC)

OHSC were obtained from seven to nine days (d) old Spargue-Dawley rats. After decapitation brains were dissected under aseptic conditions as previously described [14]–[16]. Frontal lobe and cerebellum were removed and the remaining brain containing the temporal lobe was transferred to the preparation medium (MEM, pH 7.35 containing 1% (v/v) glutamine). Horizontal slices of 350 µm thickness were prepared using a vibratome at 4°C. Hippocampi were separated and placed on cell-culture inserts with a pore size of 0.4 µm. Six to eight OHSC were obtained from each brain. Inserts were transferred to six-well culture dishes and 1 ml of culture medium containing 50% (v/v) MEM, 25% (v/v) HBSS, 12.5% NHS, 2% (v/v) glutamine, 1.2 mg/ml glucose, 0.1 mg/ml streptomycin, 100 mg/ml penicillin, 0.8 mg/ml ascorbic acid and 1 mg/ml insulin; pH 7.4 was added. Subsequently, culture dishes were incubated for six days at 35°C in a fully humidified atmosphere with 5% (v/v) CO2 and culture medium was changed every second day.

#### Time-series and excitotoxic-lesion of OHSC

At six days in vitro (div) OHSC were randomly allocated to control or NMDA group.

In five independent experiments slices were left untreated or excitotoxically lesioned by 50 µmol/l NMDA for 4 hours (h). OHSC of both groups were fixed or transferred to lysis buffer after 5 minutes (min; n = 21 each), 1 h (n = 18 each), 6 h (n = 15 each), 12 h (n = 18 each), 24 h (n = 21 each), 48 h (n = 24 each) and 72 h (n = 18 each), respectively. For the lesioned-group the time point zero was defined as starting point of NMDA application.

#### Permanent middle cerebral artery occlusion (pMCAO)

Spontaneously hypertensive rats (Charles River, Sulzfeld, Germany) were deeply anesthetised by intraperitoneal injection of ketamine hydrochloride (100 mg/kg), xylacin (10 mg/kg) and atropine (0.1 mg/kg). Animals were subjected to pMCAO by thermocoagulation as described previously [17], [18]. After surgery animals were randomly assigned to survival periods of 75 min, 1 d, 7 d, 10 d and 60 d (n = 3 each) after stroke onset. Animals were sacrificed at given time points by CO_2_ exposure and transcardially perfused with phosphate buffered saline solution (PBS) and formalin solution (4% (w/v)). Brains were removed, cryoprotected in 30% sucrose solution and stored at −80°C until further use.

### Analysis methods

#### Western Blot

All Western blot analyses were performed under reducing conditions. At first, hippocampi were transferred to lysis buffer solution (80 mmol/l Tris, 70 mmol/l sodium dodecylsulfate (SDS), 0.3 mol/l sucrose, 3 mmol/l sodium orthovanadate and 0.5 mmol/l phenylmethylsulfonyl fluoride (PMSF) at pH 7.4) and stored at −80°C until further use. The tissues were sonicated in lysis buffer solution and whole cell extracts were centrifuged at 3000 g for 10 min to remove cell debris. Protein concentrations in supernatants were measured by BCA test.

Subsequently, protein concentrates were treated with 4× Laemmli sample buffer (32% 0.5 mol/l Tris, 15% distilled water, 40% Glycerol, 10% of 10% (v/v) SDS, 2.8% β-mercaptoethanol, 0.2% bromophenol blue). Afterwards, equal protein quantities were loaded onto the top of 12.5% (v/v) SDS-polyacrylamide gels. After electrophoresis, proteins were electrotransferred to nitrocellulose membranes followed by incubation with blocking buffer solution (5% (w/v) milk, 25 mmol/l Tris, 150 mmol/l NaCl) pH 7.5). The membranes were incubated for 16 h with antibodies for Glo1 (1∶1000) and beta-actin (1∶40000) in blocking buffer solution containing 0.2% (v/v) Tween 20). After three washing steps with Tris buffered saline solution containing 0.05% (v/v) Tween 20 (TBST) for 10 min the secondary horseradish peroxidase anti-mouse antibody (1∶10000) were added for 1 h.

The membranes were subjected to enhanced chemiluminescence and the bands were visualised with X-ray films followed by semiquantitative analysis using the ImageJ image software (ImageJ 1.43, imagej.nih.gov/ij/). The beta-actin level was used as reference. Values obtained from the control-group were set arbitrarily to 100% and alternations in the lesioned-group were examined in relation to their corresponding time control.

#### Immunohistochemistry

OHSC were fixed with 4% (w/v) paraformaldehyde solution in 0.2 mol/l phosphate buffer (PB) for at least 12 h. Subsequently, OHSC were incubated in 10% (w/v) sucrose followed by 20% (w/v) and 30% (w/v) sucrose, each step were performed for at least 24 h. Afterwards, 12 µm thick sections were prepared by using a cryostat and mounted on superfrost microscope slides. Cryopreserved brains from pMCAO-experiments were cut in 20 µm thick coronal cryosections and mounted on coated slides.

For immunofluorescence, cryosections were dried for 30 min and washed with PBS containing 3% (v/v) Triton-X 100 (PBS/Triton) for 5 min. Accordingly, sections were incubated with normal goat serum (1∶20 diluted in PBS/Triton) for 30 min at room temperature to block unspecific binding sites. Subsequently, following primary antibodies were added in PBS/Triton containing 0.5% (w/v) bovine albumin serum for at least 16 h by 4°C: Glo1 (1∶150), NeuN (1∶500), GFAP (1∶400), laminin (1∶200), N-Cadherin (1∶100) or Iba1 (1∶500). Sections were then washed three times for 10 min with PBS/Triton followed by the incubation with Alexa 488-conjugated goat anti-mouse IgG (1∶200) and Alexa 568- conjugated goat anti-rabbit IgG (1∶500), respectively. Slides were then coversliped with Dako fluorescent mounting medium, and analysed by a Zeiss LSM 510 Meta confocal laser scanning system, as previously described [19], [20]. For ExtrAvidin peroxidase staining, sections were treated with methanol containing 1.5% (v/v) of 30% (v/v) hydrogen peroxide for 10 min. Afterwards, slides were washed three times for 10 min with PBS/Triton followed by incubation with normal goat serum (1∶20 in PBS/Triton X-100) for 30 min at room temperature. Subsequently, sections were incubated with Glo1 antibody (1∶300) in PBS/Triton containing 0.5% (w/v) bovine albumin serum for at least 16 h at 4°C. Afterwards, slides were washed three times for 10 min with PBS/Triton and then incubated with biotinylated goat anti-mouse IgG (1∶100) in PBS/Triton X-100 for 60 min. Next, sections were washed again three times for 10 min with PBS/Triton X-100 followed by adding of ExtrAvidin-Horseradish Peroxidase (1∶100) in PBS/Triton X-100. To visualize the immunoreactivity the chromogen 3.3-diamino-benzidine was used. Finally, slides were coversliped with Entellan.

#### Statistical analysis

Data from at least three independent experiments are presented as mean ± standard error of the mean (SEM).

Statistic analysis were performed using one and two way ANOVA followed by Bonferroni posttest, p<0.05 was considered as significant.

Graphs were blotted and analyses were arranged using Graph Pad Prism software 5 (GraphPad software, La Jolla, USA).

## Results

### Glo1 is temporally regulated after excitotoxic lesion

**Figure 1:**
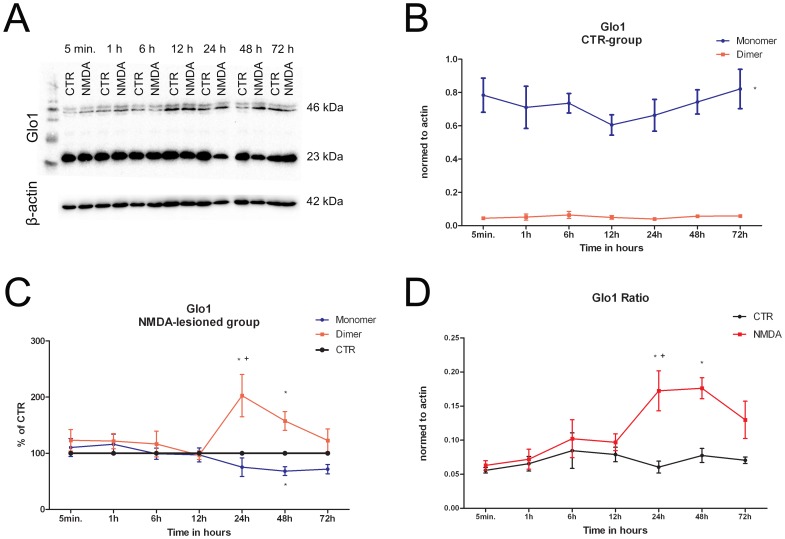
Time-dependent protein changes of Glo1 in control- and excitotoxically– lesioned OHSC from rats. **A** Representative Western blot of Glo1 and the related β-actin signals for standardisation. Glo1 antibody marked two bands: one with a molecular mass of 23 kDa referring to the monomer and a second with a molecular mass of 46 kDa representing the dimer of Glo1. **B** Temporal dynamics of Glo1 (monomer, dimer) in control OHSC normalised to their related β-actin values. No significant changes for Glo1 monomer and dimer were found over the examined time frame. However, at all investigated time points significant differences between monomer and dimer levels were present (p<0.0001). **C** Alterations of Glo1 (monomer, dimer, CTR) after excitotoxically-lesion in a time-dependent manner as normalised against β-actin values. Subsequently, monomer and dimer levels of the lesioned-group were normalised and presented in relation to their respective time controls which were set arbitrarily to 100%. The Glo1 monomer showed a significant difference at 48 h compared to time control (*, p<0.05). Statistically differences for the Glo1 dimer were found at 24 h and 48 h post-injury (*, 24 h, p<0.0001; 48 h, p<0.001) compared to monomer and time control. Furthermore, a significant increase in dimer levels was determined from 12 h to 24 h after NMDA-application (+, p<0.05). **D** The alteration of the Glo1 dimer/monomer (ratio) in OHSC as a time-dependent phenomenon of excitotoxicty. We observed a significant increase in the ratio of NMDA-group from 1 h to 24 h after NMDA-treatment (+, p<0.05). Moreover, significant differences between control- and NMDA-group were found at 24 h and 48 h after NMDA exposition (*, p<0.0001).

The antibody against Glo1 marked two bands with a molecular mass of 23 kDa and 46 kDa (Figure 1A), respectively, representing the monomer and dimer of the enzyme [2].

To analyse a possible alteration of the dimer/monomer ratio, both protein bands were determined semi-quantitatively at indicated time points (Tab S1).

In the control-group the Glo1 monomer (CTR, Figure 1A) showed constant levels up to 6 h followed by a slight decrease of intensity. Subsequently, the monomer increased up to 72 h. However, all variations did not achieve the level of significance (5 min, 1 h, 6 h, 12 h, 24 h, 48 h and 72 h, p>0.05, Figure 1B, Table S1A). The Glo1 dimer remained constant at all-time points investigated (p>0.05). Statistical analysis between monomer and dimer at their corresponding time values were statistically significant at all investigated time points (p<0.0001).

For analyses of the groups after the excitotoxic lesion intensity values were normalized and presented in relation to their respective time controls, which were set to 100% (Figure 1C). For the Glo1 monomer no significant changes of the protein level were found (p>0.05, Table S1B). Comparing monomer- and control-values revealed a significant decrease for the Glo1 monomer at 48 h (p<0.05). Values for Glo1 dimer showed no significant changes between 5 min to 12 h (p>0.05). From 12 h to 24 h we measured a significant increase in intensity (p<0.05) followed by a progressively decrease of the protein level from 24 h to 72 h. In addition, significant changes were observed for monomer at 24 h and dimer values at 24 h and 48 h when compared to respective time controls (p<0.05, Figure 1C).

Furthermore, changes of Glo1 monomer/dimer-ratios were investigated by analysing their values in control and NMDA-treated groups (Figure 1D). Examining Glo1 ratio of control-OHSC yielded no significant changes at all-time points investigated (p>0.05). Within the NMDA-lesioned group the Glo1 ratio became statistically significant at 24 h (p<0.05). The comparison between ratio values of controls and NMDA-treated slices revealed a significant increase in intensity at 24 h and 48 h (p<0.0001).

### Excitotoxic neuronal lesion triggers redistribution of Glo1

**Figure 2:**
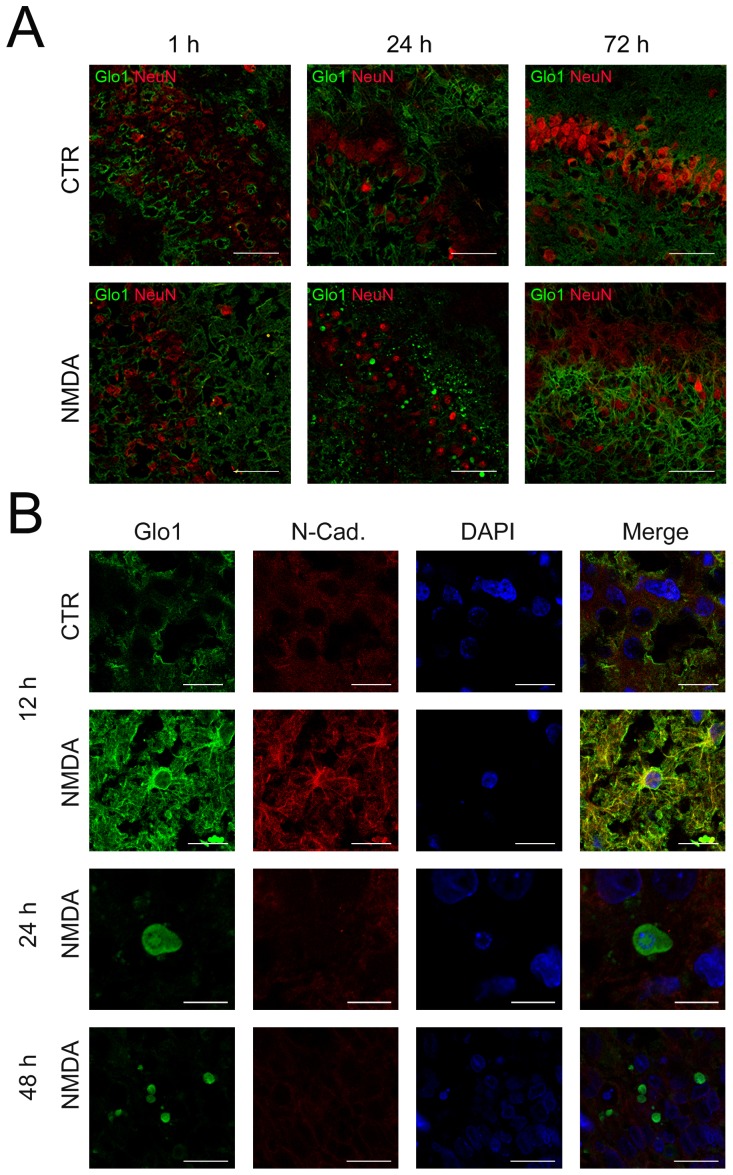
Morphological Glo1 variations after NMDA-treatment. **A** OHSC immunostained with Glo1 and NeuN. Appearance of small round Glo1 positive cells 24-application. These cells disappeared at 72 h and only astrocytic processes displayed Glo1 immunoreactivity. Scale bar: 50 µm. **B** Translocation of Glo1 from cytosol to cell membrane in neurons 12 h after NMDA treatment. OHSC stained with Glo1, the neuronal cell membrane marker N-Cadherin and DAPI for the nuclei. Scale bar: 20 µm. In the time-frame between 24 h and 48 h small round Glo1 positive cells appeared. Over time a progressive nuclear fragmentation and reduction in size (24 h, diameter = 7.86 µm±1.48; 48 h, diameter = 4.45 µm±0.94) was observed. Scale bar: 10 µm (24 h), 20 µm (48 h).

In OHSC, the localisation and cellular allocation of Glo1 in the dentate gyrus of OHSC were examined by double-immunostaining with NeuN (Figure 2A), GFAP, Iba1 combined with N-Cadherin (Figure 2B). In non-lesioned OHSC no Glo1 immunoreaction was present in NeuN immunoreactive neurons (Figure 2A). Until 12 h post ictus no alterations of Glo1 immunoreaction were observed in neurons of lesioned-OHSC as well. Double staining of Glo1 with GFAP or Iba1 revealed Glo1 immunoreactivity in astrocytes and microglia in control- and lesioned-OHSC (data not shown). Differences over the time were not present. At 24 h and 48 h post-lesion small (24 h, diameter = 7.86 µm±1.48; 48 h, diameter = 4.45 µm±0.94) round Glo1 positive, NeuN-negative cells with karyorrhectic nuclei which were noticed (Figure 2A). These immunoreactions disappeared at 72 h post-lesion. Only astrocytic processes were found immunoreactive for Glo1 at this time point.

At higher magnification a change in the distribution of Glo1 immunoreactivity was observed in neurons. Twelve hours after the lesion a translocation of Glo1 from cytosol to the cell membrane was noticed by double labelling with Glo1 and N-Cadherin, a neuronal cell membrane marker that was not found in the control-group. Moreover, a reduction in size and a progressive fragmentation of the nuclei of the small round Glo1 positive cells were detected (Figure 2B).

### Temporal and spatial dynamics of Glo1 after brain ischemia

**Figure 3:**
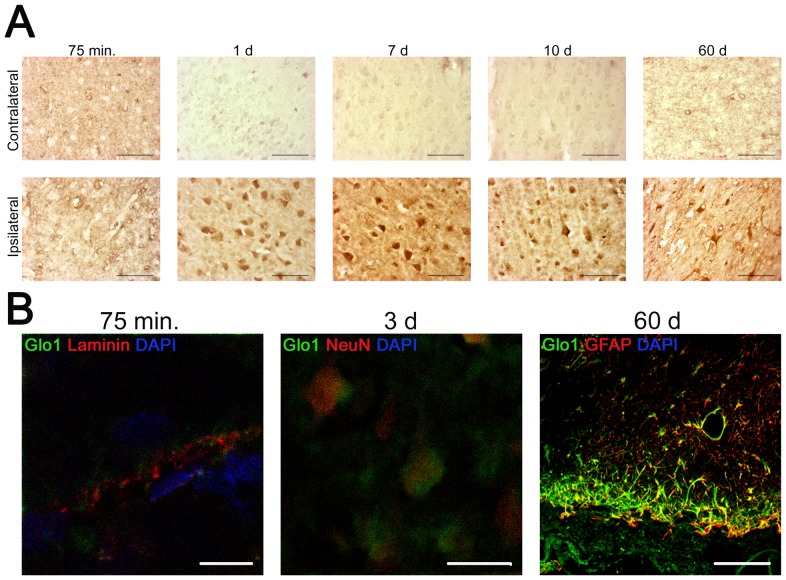
Glo1 is expressed in different cell types and underlies a time-dependent changes in immunoreactivity after pMCAO. **A** Coronal sections of cerebral cortex of spontaneously hypertensive rats with pMCAO were stained for Glo1. Immunohistochemistry shows the injury- and time-dependent Glo1 immunoreactivity in the different stages of secondary neuronal injury. **B** Identification of Glo1 immunoreactive cells. Sections were subjected to immunofluorescence for Glo1, DAPI for the nuclei and typical markers for endothelial cells (75 min; laminin), neurons (3 d; NeuN) or astrocytes (60 d; GFAP). Scale bars: 300 µm (**A**), 10 µm (**B; 75 min**), 20 µm (**B; 3 d**) and 100 µm (**B; 60 d**).

After pMCAO the contralateral site served as the control-site and the ipsilateral site were examined for injury-dependent reactions. Glo1 immunoreactivity was observed in the endothelium of blood vessels at all time points investigated (Figure 3A). In comparison to the contralateral site Glo1 immunoreaction increased 75 min after ischemia in the endothelium of blood vessels. In the time frame between 1 d and 10 d after injury high Glo1 immunoreactivity was present in neurons at the ipsilateral site. At 60 d post-injury the Glo1 immunoreactivity was not detectable in neurons, but observed in perilesional astrocytes.

Double labelling experiments with classical markers for endothelium (laminin), neurons (NeuN) and astrocytes (GFAP) (Figure 3B) corroborated our data from immunohistochemistry. At 75 min after the onset of ischemia a clear endothelial localization on contra- and ipsilateral sites with a higher intensity on ipsilateral site was detected in sections stained for laminin, Glo1 and DAPI endothelium of blood vessels showed. Sections stained with NeuN, Glo1 and DAPI showed 3 d post-injury a positive immunoreaction especially in neurons of the ischaemic core. On the contralateral site no Glo1 positive neurons were found. Sixty days after pMCAO, astrocytes participating in the formation of the ischemic scar on the ipsilateral site were identified as the major source for Glo1 in sections stained for GFAP, Glo1 and DAPI. The reaction was confined to the ipsilateral site.

## Discussion

Beside heart diseases and cancer, stroke is the most frequent cause of death and permanent disability in adulthood [21]. The only effective and accepted therapy is the early thrombolysis if started in the first 4.5 h after ischemia [22].

The illumination of processes in the aftermath of the ischemic brain injury and the inhibition of downstream events after ischemia are promising approaches to diminish secondary neuronal damage. The activation of NMDA-receptor due to high releases of glutamate after and during ischaemic injury results in an excitotoxic insult [23]. In animal studies inhibition of NMDA-receptor improved the neurological score and reduced the infarct volume [24] but so far in clinical trials no positive effects were reached [21]. The relevance of calcium-influx to mitochondria [25] has extensively been investigated and the thereby related mitochondrial-dysfunction with caspase-3 activation [26] and ROS-production [27], [28] are believed to maintain apoptosis or necrosis [29]. Changes in glucose concentration markedly affect the survival of neuronal tissue. Recurrent glucose deprivation was found to result in higher neuronal loss [30]. The inhibition of hexokinase, the rate-limiting enzyme of glycolysis showed protective effects after excitotoxicity [31]. The inhibition of Glo1 leads to enhanced MG levels and prolonged induction of caspase-3 activation and alterations in calcium homeostasis as shown in human neuroblastoma cells SH-SY5Y [32], [33]. Higher concentrations of MG attenuated cell viability of hippocampal and cortical neurons [8], [34]. Furthermore, increasing MG levels were paralleled by enhanced mitochondrial dysfunction, ROS, IL-1β, and TNFα production, DNA fragmentation, GSH depletion, modifications of transcription factors and signal transductions being essential for neurorepair [6], [8], [9], [34], [35], [36]. However, little is known about the role and temporal dynamics of Glo1 in secondary neuronal lesions.

In the present study we examined the role of Glo1 by Western blot analyses and immunohistochemistry in an in vitro model of excitotoxically-lesioned OHSC and in the in vivo model of pMCAO.

Glo1 is a dimeric enzyme which consists of two homologous monomers which are non covalently bond [2]. The presence of Glo1 dimers after the injury suppose a modification of Glo1 leading to Glo1 dimerization. It is well accepted, that excessive NMDA receptor activation results in an excessive formation of NO [38]. High NO levels and nitrosylsation of proteins contribute to neuronal damage as shown in the model of cerebral ischemia [39], [40]. Glo1 is a NO-responsive protein and nitrosylation decreases Glo1 activity [41], [42]. After nitrosylation Glo1 might also be phosphorylated at threonine-106 and is subsequently no longer involved in detoxification of MG. Interestingly, the modified Glo1 form suppresses TNF-induced transcriptional activity of NF-κB reporter gene accompanied by increased cell viability [43]. However, it has to be mentioned that nitrosylation is also affected and thereby resolved using reducing conditions which contains SDS and mercaptoethanol [44]. Recently, we have shown the impairment of Glo1 activity by glutahionylation as another posttranslational modification [45]. In addition, other groups reported on changes of Glo1 dimer or oligomer stability by glutathionylation which was paralleled by dissociation and decreased protein function [46], [47], [48], [49]. Data about posttranslational modifications leading to Glo1 dimerization and their involvement and function in neuronal injury are still missing. A possible link might be calcium dependent covalent cross-links by transglutaminases, that showed increased activity in the aftermath of NMDA treatment [50], [51], [52]. Since dimer formation related to nitrosylation, glutathionylation or disulfid bridges can be excluded under reducing conditions, the nature of the observed crosslink remains to be determined. Further mass spectrometric analysis will be necessary to bring more light in this issue.

In the control-group time dependent variations of Glo1 monomer were observed presumably representing intrinsic rhythms as present in most physiological systems. In response to NMDA application Glo1 dimer levels strongly increased between 12 h and 24 h post injury followed by a progressive decrease to control levels. Looking at temporal dynamics of glyoxalase 1 and its ratio, a significant rise from 12 h to 24 h was observed indicating an increase in Glo1 dimer after excitotoxicity paralleled by a decrease of the monomer. Taking previous studies into consideration, the increase in Glo1 levels might presumably be a part of survival mechanisms within the cells to prevent excitotoxic death [42], [43]. Regarding the redistribution of Glo1 immunoreaction in neurons from cytosol to cell membrane 12 h after lesion there are no reports in the literature and its possible functional relevance remains unknown. Nevertheless, the paralleled increase of Glo1 dimer and the translocation of immunoreaction may indicate a new function of Glo1 in lesioned neurons. Furthermore, Glo1 up-regulation in astrocytes was observed 72 h after NMDA application. This shift of immunoreactivity might represent the necessity for astrocytic Glo1 up-regulation. Recent results about the neuronal vulnerability toward changes of glucose metabolism affirm the relevance of astrocytic Glo1 in metabolic homeostasis presumptive after neuronal injury and increased MG exposition to protect surrounding neurons of oxidative stress [10].

Upon MG accumulation on cellular level nuclear fragmentation, formation of DNA-ladder and apoptosis based on capase-3 activation have been reported [9], [36], [32], [5], [53]. Alternatively, the increased Glo1 immunoreactivity in astrocytes may indicate a metabolic turnover to assist neurons because of their low capacity to change their metabolism upon oxidative stress [10]. Both processes might contribute to neuroprotection under stress.

Based on previously described deficits of glucose metabolism after traumatic brain injury [54] and excitoxicity as an accompanying event of secondary neuronal injury after stroke [24], [37] we conducted pMCAO as a well established in vivo model. In immunohistochemistry we observed Glo1 immunoreactivity only on the ipsilateral site with an apparent change of Glo1 expressing cells. Immunofluorescence analysis with classical markers for endothelium (laminin), neurons (NeuN) and astrocytes (GFAP) in vivo support our findings of in vitro experiments. Under physiological conditions the endothelium of blood vessels showed Glo1 immunoreactivity on the contra- and ipsilateral sites. The immunoreaction became more intense at the ipsilateral site 75 min after injury. In the following, neurons showed the strongest Glo1 immunoreaction from 1 d to 10 d after the onset of ischaemia. Sixty days after vessel occlusion no more Glo1-positive neurons were noticed. In contrast, astrocytes near the scar displayed valuable amounts of Glo1. However, the role of Glo1 in scar formation needs to be elucidated further. We assume an elevated metabolic flux in endothelial cells by the hypoxic state after the occlusion to save or restore the integrity of the blood brain barrier. The upregulation and redistribution of Glo1 might be a consequence to prevent MG-induced cell death.

## Conclusions

Our data reveal time-dependent and injury-related variations of Glo1 expression and cellular localization in the aftermath of excitoxicity and pMCAO. The rearrangement of Glo1 between its dimer and monomer as well as the cell-type specific time points of temporal Glo1 expression (75 min, endothelium; 1 d–10 d, neurons; 60 d, astrocytes) might help to determine the onset of ischemia and clarify the accompanied metabolic imbalance after acute neuronal lesion. However, the role of the Glo1 dimer and Glo1 translocation has to be illuminated further.

## Supporting Information

Table S1 A: Values from Western Blot analysis for the control-group normalised to β-actin. B: Values from Western Blot analysis for the NMDA-lesioned group normalised to corresponding time controls. C: Generated Glo1 ratio for control- and NMDA-lesioned group normalised to β-actin.
(DOC)
