# Supplementary material for: Temporal Dynamics of Glyoxalase 1 in Secondary Neuronal Injury
Source: PLoS One. 2014 Feb 3;9(2):e87364. doi: 10.1371/journal.pone.0087364 (PMC3911945; doi:10.1371/journal.pone.0087364)
Supplement: Table S1 — A: Values from Western Blot analysis for the control-group normalised to β-actin. B: Values from Western Blot analysis for the NMDA-lesioned group normalised to corresponding time controls. C: Generated Glo1 ratio for control- and NMDA-lesioned group normalised to β-actin. (DOC) [file pone.0087364.s001.doc]

**Supporting Information Legends:**

**Table S1**

A: Values from Western Blot analysis for the control-group normalised to β-actin

| Time | Monomer | Dimer |  |
| --- | --- | --- | --- |
| 5 min. | 0.78±0.10 | 0.04±0.01 | p<0.0001 |
| 1 h | 0.71±0.13 | 0.05±0.02 | p<0.0001 |
| 6 h | 0.74±0.06 | 0.06±0.02 | p<0.0001 |
| 12 h | 0.61±0.06 | 0.05±0.01 | p<0.0001 |
| 24 h | 0.66±0.10 | 0.04±0.01 | p<0.0001 |
| 48 h | 0.74±0.07 | 0.06±0.01 | p<0.0001 |
| 72 h | 0.82±0.12 | 0.06±0.01 | p<0.0001 |
|  | For all p>0.05 | For all p>0.05 |  |

B: Values from Western Blot analysis for the NMDA-lesioned group normalised to corresponding time controls

| Time | Monomer | Dimer |  |
| --- | --- | --- | --- |
| 5 min. | 110.30%±16.01 | 123.10%±19.14 | p>0.05 |
| 1 h | 116.00%±17.87 | 121.60%±13.63 | p>0.05 |
| 6 h | 99.01%±9.96 | 116.50%±22.94 | p>0.05 |
| 12 h | 97.06%±12.34 | 96.55%±7.61 | p<0.05 |
| 24 h | 75.18%±16.49 | *202.60%±37.53* | p<0.0001 |
| 48 h | *68.09%±7.83* | *157.50%±16.58* | p<0.001 |
| 72 h | 71.69%±8.27 | 122.70%±20.38 | p>0.05 |
|  | p<0.05 for 48 h | p<0.05, 24 h |  |

C: Generated Glo1 ratio for control- and NMDA-lesioned group normalised to β-actin

| Time | CTR- group | NMDA-group |  |
| --- | --- | --- | --- |
| 5 min. | 0.06±0.01 | 0.06±0.01 | p>0.05 |
| 1 h | 0.07±0.01 | 0.07±0.01 | p>0.05 |
| 6 h | 0.08±0.03 | 0.10±0.03 | p>0.05 |
| 12 h | 0.08±0.01 | 0.10±0.01 | p>0.05 |
| 24 h | 0.06±0.01 | *0.17±0.03* | p<0.0001 |
| 48 h | 0.08±0.01 | *0.18±0.02* | p<0.0001 |
| 72 h | 0.07±0.01 | 0.13±0.03 | p>0.05 |
|  | For all p>0.05 | p<0.05, 1 h vs. 24 h | |
